# Genetic Evidence for Possible Involvement of the Calcium Channel Gene *CACNA1A* in Autism Pathogenesis in Chinese Han Population

**DOI:** 10.1371/journal.pone.0142887

**Published:** 2015-11-13

**Authors:** Jun Li, Yang You, Weihua Yue, Meixiang Jia, Hao Yu, Tianlan Lu, Zhiliu Wu, Yanyan Ruan, Lifang Wang, Dai Zhang

**Affiliations:** 1 Institute of Mental Health, The Sixth Hospital, Peking University, Beijing, China; 2 Key Laboratory of Mental Health, Ministry of Health & National Clinical Research Center for Mental Disorders (Peking University), Beijing, China; 3 Peking-Tsinghua Center for Life Sciences, Peking University, Beijing, P. R. China; 4 PKU-IDG/McGovern Institute for Brain Research, Peking University, Beijing, P. R. China; The George Washington University, UNITED STATES

## Abstract

Autism spectrum disorders (ASD) are a group of neurodevelopmental disorders. Recent studies suggested that calcium channel genes might be involved in the genetic etiology of ASD. *CACNA1A*, encoding an alpha-1 subunit of voltage-gated calcium channel, has been reported to play an important role in neural development. Previous study detected that a single nucleotide polymorphism (SNP) in *CACNA1A* confers risk to ASD in Central European population. However, the genetic relationship between autism and *CACNA1A* in Chinese Han population remains unclear. To explore the association of *CACNA1A* with autism, we performed a family-based association study. First, we carried out a family-based association test between twelve tagged SNPs and autism in 239 trios. To further confirm the association, the sample size was expanded to 553 trios by recruiting 314 additional trios. In a total of 553 trios, we identified association of rs7249246 and rs12609735 with autism though this would not survive after Bonferroni correction. Our findings suggest that *CACNA1A* might play a role in the etiology of autism.

## Introduction

Autism spectrum disorders (ASD) are a common neurodevelopmental disorder characterized by abnormality in three domains: deficit in social interaction, impairment in communication skills, and the presence of stereotyped behaviors [1]. These core symptoms are detected within the first three years of age. Previous studies reported that ASD affect approximately 1 out of 88 children, with a male to female ratio of 4:1 [2,3]. Autism is generally attributed strong genetic underpinnings. Twin studies suggested an estimated heritability of almost 90% for autism. The risk among siblings of affected individuals is approximately 20–80 times higher than that of the general population [3].

The calcium ion, one of the most ubiquitous biological signaling molecules, plays important roles in the central nervous system. Disruption of intracellular calcium homeostasis underlies a host of neurologic diseases such as seizures and autism [4]. A previous study indicated that neocortical calcium level in autistic patients is significantly higher than that of healthy controls [5]. In addition, postmortem analysis of brain tissues from ASD patients showed alterations of calcium signaling [6]. Moreover, animal models such as *Cd38* ^*-/-*^ and *Cadps2* ^*-/-*^ mice with calcium signaling dysfunction have autistic-like behavioral deficits, for example, reduced social interaction [7, 8]. Taken together, these studies suggest that Ca^2+^ homeostasis might play a crucial role in the pathogenesis of ASD.

Calcium channels mediate the influx of calcium ions into the cell upon membrane depolarization, and play critical roles in neuronal excitability, depolarization-evoked neurotransmitter release, and gene expression [9]. Accumulative genetic evidence has implicated the significant association between calcium channel genes and autism. A previous study reported a *de novo* mutation in the exon of *CACNA1C*, which encodes an alpha 1C subunit of voltage-gated calcium channel, in patients with Timothy syndrome [10], characterized by major developmental delays, cognitive impairments, and autism spectrum disorders.

Another study revealed that several SNPs, including SNPs in *CACNA1C* and another calcium channel gene *CACNB2*, are significantly associated with five major psychiatric disorders, including ASD [11]. Wang et al. reported suggestive evidence of an association between ASD and SNPs surrounding *CACNA1C* [12]. In addition, genome-wide association study revealed that four SNPs in three calcium channel genes (*CACNA1C*, *CACNA1G*, and *CACNA1I*) are associated with ASD [13]. These lines of evidence provide strong support for calcium channel genes as candidate genes of autism.

*CACNA1A* (calcium channel, voltage-dependent, P/Q type, alpha 1A subunit) encodes an alpha-1 subunit of voltage-gated calcium channel, is predominantly expressed in the cerebellum, frontal cortex and hippocampus. It was previously shown that the second cistron of *CACNA1A* encodes a transcription factor mediating cerebellar development [14]. Moreover, specific ablation of *Cacna1a* in mouse forebrain results in multiple cognitive impairments, and *Cacna1a* knock-out mice show deficits in spatial learning and reduced recognition memory [15]. Previous studies have demonstrated that mutations in *CACNA1A* are associated with three neurologic disorders, including familial hemiplegic migraine, epilepsy, and episodic and progressive ataxia [16,17]. *CACNA1A* is located on chromosome 19p13, and a previous study identified the region of suggestive linkage with autism on 19p13 [18]. Furthermore, a 2-year-old girl with *de novo* 19p13 deletion was shown to have severe psychomotor impairment, language delay, intellectual disability and seizures [19]. It is known that autism coexist with seizures and mental retardation in up to 30% and 80% of patients, respectively [20]. Interestingly, a SNP in *CACNA1A* was found among the top 15 SNPs contributing to ASD diagnosis prediction in a CEU (Utah residents with Northern and Western European ancestry from the CEPH collection) cohort [21]. Therefore, we hypothesized that *CACNA1A* might be involved in the etiology of autism.

We performed a family-based association study to assess the association between *CACNA1A* and autism in Chinese Han population. Our results showed association between rs12609735 and rs7249246 in *CACNA1A* with autism in a total of 553 trios though statistical significance was not reached after Bonferroni correction. These results suggest that *CACNA1A* might be a susceptibility gene for autism.

## Methods and Materials

### Ethics Statement

This study was approved by the Ethics Committee of Institute of Mental Health, The Sixth Hospital, Peking University. All subjects provided written informed consent, and informed written consent for children was obtained from their biological parents (the children’s legal guardians).

### Subjects

A total of 553 Chinese Han trios (children affected with autism and their unaffected biological parents) were assessed. By using the genetic power calculator software, we selected a cohort of 239 trios as a discovery cohort. Among the 239 autistic children, 226 were male and 13 female. Their ages at the clinical assessment ranged from 2 to 17 years, averaging 7.5 years. Then, we expanded our sample size to 553 trios (1659 individuals) by recruiting 314 additional trios (median age of 6.0 years old). Of all 553 autistic children, 513 were male and 40 female. The trios were recruited at the Institute of Mental Health, Peking University, China.

Children with autism were evaluated using the DSM-IV diagnostic criteria by two senior psychiatrists. For clinical assessment, the Autism Behavior Checklist (ABC) [22] and Childhood Autism Rating Scale (CARS) [23] were used. Children with phenylketonuria, fragile X syndrome, tuberous sclerosis, and chromosomal abnormality by karyotyping analysis were excluded. To decrease the heterogeneity of the cases, children affected with Asperger disorder and Rett syndrome were also excluded. Genomic DNA was extracted from peripheral blood of all individuals using Qiagen QIAamp DNA Kits after informed consent.

### SNP selection and genotyping

**Fig 1:**
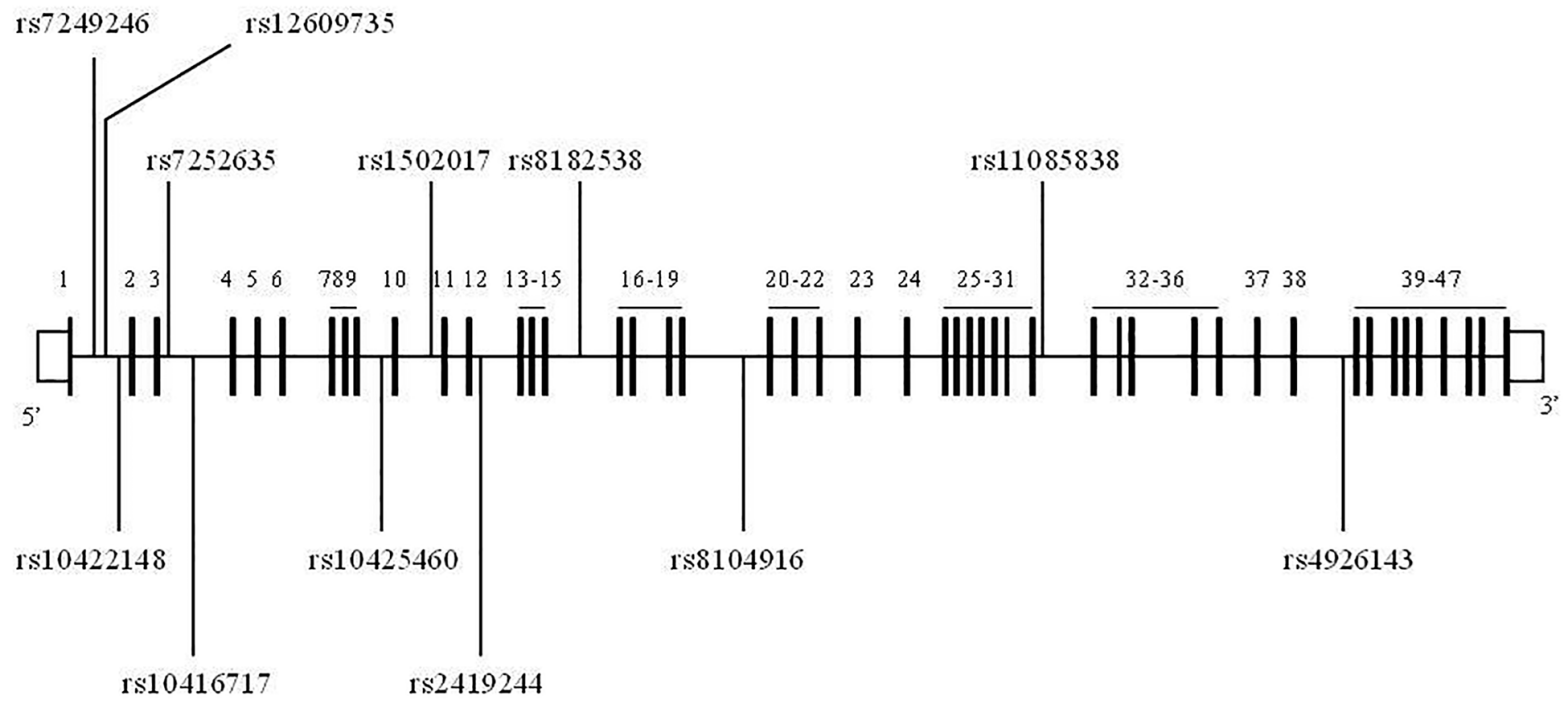
A diagram of the position of selected 12 SNPs in *CACNA1A*. A diagram of the structure of *CACNA1A*, exons are in black. The selected 12 SNPs were noted.

Genotype data in Chinese Han in Beijing (CHB) from HapMap phase Ⅱ and Ⅲ were downloaded from the HapMap genotype dataset (http://hapmap.ncbi.nlm.nih.gov/). Then, pairwise tagging in the Tagger module in Haploview version 4.2 program (http://www.broad.mit.edu/mpg/haploview/) was also carried out to select SNPs that could capture the common genetic variations (*r* ^2^>0.8). Furthermore, physical distance between SNPs and minor allele frequency (MAF)>0.05 were considered. Therefore, twelve tagged SNPs in *CACAN1A* were selected from 13214919 bp to 13488269 bp on chromosome 19 with a mean inter-SNP distance of 23 Kb (GRCH38, National Center for Biotechnology Information [NCBI]) (Fig 1).

All SNPs were genotyped using the Sequenom genotyping platform (http://www.sequenom.com/), which uses the MALDI-TOF (matrix-assisted laser desorption/ionization time-of-flight) primer extension assay. Primers were designed according to the sequence of the forward strand from the dbSNP database (http://www.ncbi.nlm.nih.gov/SNP/). The iPlex genotyping assay was used, which resulted in increased plexing efficiency and flexibility for the MassARRAY system through single base primer extension with mass-modified terminators [24,25].

### Statistical analysis

The Hardy-Weinberg Equilibrium (HWE) for genotype frequency distributions was tested using the chi-square goodness-of-fit test. Mendelian inconsistencies were tested using the family-based association test (FBAT) software (http://www.biostat.harvard.edu/~fbat/default.html). Genotypes of families with Mendelian errors were reset to zero. We used Haploview version 4.2 to calculate the pairwise *D'* values for SNPs and construct haplotypes. SNP pairs were considered to be in strong linkage disequilibrium (LD) if *D'*>0.7.

Association analyses for SNPs were performed using FBAT v1.7.2 [26]. The inheritance models examined were based on the number of copies of an allele required for increased susceptibility, and included additive, dominant, and recessive models, defined as follows: (1) additive, 1 or 2 copies of a risk allele increase the likelihood of possessing a trait in an additive fashion (i.e., risk with 2 alleles >1 allele >0 allele), (2) dominant, 1 or 2 copies of an allele are associated with an equal likelihood of having a trait, and (3) recessive, 2 copies of an allele are necessary to increase the likelihood of having a trait. The inheritance modes of ASD are unclear. Therefore, the three models were examined. It should be noted that because each SNP is biallelic, the results for the recessive model is the same as the dominant model (the Z-scores for the alleles are flipped and in the opposite direction). To decrease type I error, Bonferroni correction was used, setting the significance level to *p*<0.0042 (α/n = 0.05/12 = 0.0042).

The FBAT program uses generalized score statistics to perform a variety of transmission disequilibrium tests (TDT), including haplotype analyses. individual haplotype tests were conducted under the “biallelic” mode in haplotype based association test (HBAT). Meanwhile, the global haplotype tests of association were performed under “multiallelic” mode in HBAT. Permutation tests were used for multiple testing corrections of haplotype analyses (n = 10,000).

The power for this association study was calculated using the Quanto software, version 1.2.4 (http://biostats.usc.edu/software).

### *In silico* analysis of noncoding variants and expression pattern of CACNA1A

Two software programs were used to explore the annotations of the noncoding variants. HaploReg v3 (http://compbio.mit.edu/HaploReg) was employed to identify chromatin states, conservation and regulatory motif alterations of risk variants and SNPs [27]. In addition, is-rSNP (http://bioinformatics.research.nicta.com.au/software/is-rsnp/) was used to predict whether a SNP is a regulatory SNP and the set of transcription factors (TFs) with affected binding [28]. Moreover, expression level of *CACNA1A* in different brain regions and different cell lines were measured with Gene Enrichment Profiler tool (http://xavierlab2.mgh.harvard.edu/EnrichmentProfiler/index.html) [29]. Dynamic *CACNA1A* expression along the entire development and adulthood in the cerebellar cortex, mediodorsal nucleus of the thalamus, striatum, amygdala, hippocampus, and 11 areas of neocortex were assessed via the Human Brain Transcriptome database (http://hbatlas.org/pages/hbtd) [30].

## Results

All selected 12 SNPs in *CACNA1A* were successfully genotyped. The genotype concordance rate in re-genotyped samples by Sequenom was more than 99%. Genotype frequencies and Hardy-Weinberg equilibrium data in the 239 trios are shown in S1 Table.

For the 12 SNPs with risk allele frequencies ranging from 0.276 to 0.911 in the 239 trios, the power to detect these risk alleles ranged from 37 to 87%. The power to detect the risk allele frequencies for rs7249246, rs12609735 and rs2419244 was about 87%, increasing to more than 90% after expanding the sample size to 553 trios.

In 239 trios, univariate (single marker) FBAT demonstrated that allele C of rs12609735 showed a preferential transmission from parents to autistic children under an additive model (C>T, Z = 2.261, *p* = 0.024; S2 Table). Under a recessive model, the C allele of rs12609735 showed a preferential transmission (rs12609735: C>T, Z = 2.008, *p* = 0.045). Meanwhile, the G allele of rs7249246 and the A allele of rs2419244 were under-transmitted from parents to offspring (rs7249246: G>T, Z = -2.276, *p* = 0.023; rs2419244: A>G, Z = -2.039, *p* = 0.041) (S3 Table). Results for the dominant model was the same as that of recessive model (the Z-scores for the alleles are flipped and in the opposite direction) (S4 Table)

Then, we expanded the sample size to 553 trios (1659 individuals) to further investigate the association of three SNPs (rs7249246, rs12609735, and rs2419244) with autism. The genotype distributions of SNPs in autistic children did not deviate from Hardy-Weinberg equilibrium except for rs7249246 with *p* < 0.05. However, the genotype distribution of rs7249246 in parents did not deviate from Hardy-Weinberg equilibrium (S5 Table).

Under an additive model, univariate FBAT still demonstrated that the C allele of rs12609735 showed a preferential transmission from parents to children affected with autism in the 553 trios (C>T, Z = 1.998, *p* = 0.046; Table 1). Furthermore, the G allele of rs7249246 showed an under-transmission (G>T, Z = -2.668, *p* = 0.008) under a recessive model (Table 2). The Z-scores for the alleles calculated by FBAT under the dominant model are flipped and in the opposite direction (Table 3). However, the statistical sigficance would not remain after Bonferroni correction.

**Table 1:** Results of association analyses between 3 SNPs in *CACNA1A* in 553 trios by FBAT under an additive model.

Marker	Allele	Afreq	Fam	S	E (S)	Var (S)	Z	*p*
rs7249246	G	0.500	388	372.00	381.50	128.75	-0.837	0.402
	T	0.500	388	404.00	394.50	128.75	0.837	0.402
rs12609735	C	0.343	356	306.00	284.50	115.75	1.998	**0.046**
	T	0.657	356	406.00	427.50	115.75	-1.998	**0.046**
rs2419244	A	0.561	394	423.00	433.00	129.50	-0.879	0.380
	G	0.439	394	365.00	355.00	129.50	0.879	0.380

Afreq, allele frequency; Fam, number of informative families; S, test statistics for the observed number of transmitted alleles; E(S), expected value of S under the null hypothesis (i.e., no linkage and no association).

**Table 2:** Association results between 3 SNPs in *CACNA1A* and autism in 553 trios by FBAT under a recessive model.

Marker	Allele	Afreq	Fam	S	E (S)	Var (S)	Z	*p*
rs7249246	G	0.500	251	74.00	93.75	54.81	-2.668	**0.008**
	T	0.500	264	90.00	100.25	58.06	-1.345	0.179
rs12609735	C	0.343	160	60.00	53.25	33.31	1.169	0.242
	T	0.657	303	110.00	124.75	69.06	-1.775	0.076
rs2419244	A	0.561	298	105.00	118.00	66.75	-1.591	0.112
	G	0.439	220	76.00	79.00	47.25	-0.436	0.663

Afreq, allele frequency; Fam, number of informative families; S, test statistics for the observed number of transmitted alleles; E(S), expected value of S under the null hypothesis (i.e., no linkage and no association).

**Table 3:** Association results between 3 SNPs in *CACNA1A* and autism in 553 trios by FBAT under a dominant model.

Marker	Allele	Afreq	Fam	S	E (S)	Var (S)	Z	*p*
rs7249246	G	0.500	264	174.00	163.75	58.06	1.345	0.179
	T	0.500	251	177.00	157.25	54.81	2.668	**0.008**
rs12609735	C	0.343	303	193.00	178.25	69.06	1.775	0.076
	T	0.657	160	100.00	106.75	33.31	-1.169	0.242
rs2419244	A	0.561	220	144.00	141.00	47.25	0.436	0.663
	G	0.439	298	193.00	180.00	66.75	1.591	0.112

Afreq, allele frequency; Fam, number of informative families; S, test statistics for the observed number of transmitted alleles; E(S), expected value of S under the null hypothesis (i.e., no linkage and no association).

The two SNPs rs7249246 and rs12609735 were in one LD block (*D*’ = 0.74) as shown in S1 Fig. Haplotype analysis was carried out only for the LD block. We found no significant association of the haplotypes constructed from rs7249246 and rs12609735 with autism (Table 4). Genotype data for the 239 trios and additional 314 trios are shown in S6 Table and S7 Table, respectively.

**Table 4:** Association of haplotypes constructed from rs7249246 and rs12609735 in *CACNA1A*.

Marker	Haplotypes	freq	Fam	S	E (S)	Var (S)	Z	*p*	Global *p*	Permutation^a^ *p*
rs7249246- rs12609735	G-T	0.456	355.0	352.30	367.42	112.48	-1.422	0.155	0.210	0.187
	T-C	0.299	299.8	262.30	246.92	100.15	1.541	0.123		
	T-T	0.200	285.7	191.70	197.58	82.23	-0.652	0.514		
	G-C	0.045	85.5	51.70	46.08	18.90	1.284	0.199		

^a^ Whole marker permutation test using chisq sum *p* value, the number of permutation is 10,000; freq, Estimation of haplotype frequencies; Fam, number of informative families; S, test statistics for the observed number of transmitted alleles; E(S), expected value of S under the null hypothesis (i.e., no linkage and no association).

*In silico* analysis of function prediction for noncoding variants revealed that rs7249246 might alter six motifs (S8 Table). In addition, rs7249246 and rs12609735 might be regulatory SNPs, which affect the ability of a transcription factor to bind to DNA (S9 Table). Expression pattern showed that *CACNA1A* is highly expressed in the human brain, especially the cerebellum, cerebellum peduncles and hippocampus (S2 Fig). During the developmental stages, *CACNA1A* expression level increases rapidly before the postnatal 500 days and is maintained at a relative high level stably in different brain regions throughout life (S3 Fig).

## Discussion

In the present study, we performed a family-based association study to investigate the association between *CACNA1A* and autism in Chinese Han population. Our results indicated that rs7249246 and rs12609735 in *CACNA1A* were significant associated with autism in 553 nuclear families though the association was not significant after Bonferroni correction

We observed a deviation from Hardy-Weinberg equilibrium in the affected individuals at rs7249246, while the genotype distribution of rs7249246 did not deviate from Hardy-Weinberg equilibrium in parents. This could represent a potentially interesting disease association, since the deviation was observed only in affected individuals.

*CACNA1A* is highly expressed in the cerebellum and abundant in the cerebral cortex and hippocampus throughout life. In the first three years of human life, *CACNA1A* expression levels increase rapidly in the above brain regions. Strong evidence indicate that CACNA1A significantly affects the development of the cerebellum and frontal cortex function, maintaining the balance of excitatory/inhibitory inputs [15], and gene transcription [14]. CACNA1A dysfunction might lead to cerebellar atrophy [14] and functional deficits of the cerebellum, which usually are detected in autistic patients [31–34]. In addition, more than thirty mutations in *CACNA1A* could cause deficits in neuronal transmission [35,36]. Individuals carrying *CACNA1A* mutations were shown to present congenital ataxia, cerebellar atrophy and developmental delay, sharing some clinical features of autism [37,38]. Moreover, forebrain specific *CACNA1A* knock-out mice showed impaired synaptic transmission at hippocampal glutamatergic synapses, deficits in spatial learning, reduced recognition memory, and reduced anxiety-like behaviors [15]. Some of these impairments could mimic the clinical symptoms of autism.

Multiple studies have suggested that calcium channel dysfunction might contribute to the pathogenesis of autism. Previous genetic studies have indicated an association between autism and *CACNA1C* [11–13]. A recent study identified rare causal variants of *CACNA1F* (an X-linked gene) in ASD patients by resequencing of functional genomic regions [39]. Moreover, a whole exome resequencing study identified rare *de novo* alleles of *CACNA1D* and *CACNA1E* as "top *de novo* risk mutations" for autism [40]. The calcium channel is involved in calcium-modulated functions, including intracellular signaling, neurotransmitter release and gene expression. Replicated association studies focus on calcium channel genes and functional assays of the linked polymorphisms may further expand our understanding of the relationship between autism and calcium channel function.

To decrease population stratification, we performed a family-based association study. Although a previous study suggested a SNP in *CACNA1A* to be among the top 15 SNPs contributing to the ASD diagnosis prediction in a CEU cohort [21], these results have not been confirmed by replicated research. Here we reported the association between *CACNA1A* and autism in Chinese Han population for the first time. Our study indicated that calcium channel genes might be considered susceptibility genes for autism.

In summary, we detected an association of rs7249246 and rs12609735 in *CACNA1A* with autism though this would not survive after Bonferroni correction. Our study indicated that *CACNA1A* might play a role in the pathogenetic mechanism of autism. These findings should be confirmed by additional larger sample size studies. Further mutation screening and researches on *CACNA1A* function are essential to understand the underlying mechanisms of this gene on autism.

## Supporting Information

S1 Fig The linkage disequilibium (LD) block of three SNPs in *CACNA1A* in the 553 trios. Solid spine of LD, *D*’>0.7; Markers with LD (*D*’<1 and LOD>2) are shown in pink. Regions of low LD and low LOD scores (*D*’<1 and LOD<2) are shown in white.
(DOC)

S2 Fig Enrichment profile of CACNA1A in human various cells/tissues. (DOC)

S3 Fig Dynamic expression levels of CACNA1A in the human brain throughout life. CBC, the cerebellar cortex; MD, mediodorsal nucleus of the thalamus; STR, striatum; AMY, amygdala; HIP, hippocampus; NCX, 11 areas of neocortex
(DOC)

S1 Table Information of the selected 12 SNPs in *CACNA1A* and genotype frequencies in 239 autism trios of Han Chinese descent. ^a^ Hardy-Weinberg equilibrium *p* value for genotype distributions in children affected with autism; ^b^ Hardy-Weinberg equilibrium *p* value for genotype distributions in parents.
(DOCX)

S2 Table Results of association analyses between 12 SNPs in *CACNA1A* and autism in 239 trios by FBAT under an additive model. Afreq, allele frequency; Fam, number of informative families; S, test statistics for the observed number of transmitted alleles; E(S), expected value of S under the null hypothesis (i.e., no linkage and no association).
(DOCX)

S3 Table Results of association analyses between 12 SNPs in *CACNA1A* and autism in 239 trios by FBAT under a recessive model. Afreq, allele frequency; Fam, number of informative families; S, test statistics for the observed number of transmitted alleles; E(S), expected value of S under the null hypothesis (i.e., no linkage and no association).
(DOCX)

S4 Table Results of association analyses between 12 SNPs in *CACNA1A* and autism in 239 trios by FBAT under a dominant model. Afreq, allele frequency; Fam, number of informative families; S, test statistics for the observed number of transmitted alleles; E(S), expected value of S under the null hypothesis (i.e., no linkage and no association).
(DOCX)

S5 Table Information of 3 SNPs in *CACNA1A* and genotype frequencies in 553 autism trios of Han Chinese descent. ^a^ Hardy-Weinberg equilibrium *p* value for genotype distributions in children affected with autism; ^b^ Hardy-Weinberg equilibrium *p* value for genotype distributions in parents.
(DOCX)

S6 Table Genotype data of 12 SNPs in 239 trios of Chinese decent. (XLS)

S7 Table Genotype data of 3 SNPs in additional 314 trios of Chinese decent. (XLS)

S8 Table Functional annotation for rs7249246. (XLS)

S9 Table Results of is-rSNP in predicting the effect of rs7249246 and rs12609735 on transcription factors. *p-values adjusted by Benjamini-Hochberg correction;Only BH corrected *p*<0.05 are listed.
(XLS)
