# Supplementary material for: Genetic Evidence for Possible Involvement of the Calcium Channel Gene CACNA1A in Autism Pathogenesis in Chinese Han Population
Source: PLoS One. 2015 Nov 13;10(11):e0142887. doi: 10.1371/journal.pone.0142887 (PMC4643966; doi:10.1371/journal.pone.0142887)
Supplement: S1 Fig — Solid spine of LD, D’>0.7; Markers with LD (D’<1 and LOD>2) are shown in pink. Regions of low LD and low LOD scores (D’<1 and LOD<2) are shown in white. (DOC) [file pone.0142887.s001.doc]

**S1 Fig. The linkage disequilibium (LD) block of three SNPs in *CACNA1A* in the 553 trios**


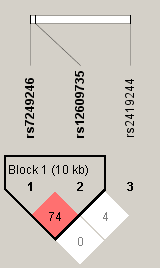


Solid spine of LD, *D*’>0.7; Markers with LD (*D*’<1 and LOD>2) are shown in pink. Regions of low LD and low LOD scores (*D*’<1 and LOD<2) are shown in white.
