# Supplementary figures and images for: Genetic Evidence for Possible Involvement of the Calcium Channel Gene CACNA1A in Autism Pathogenesis in Chinese Han Population
Source: PLoS One. 2015 Nov 13;10(11):e0142887. doi: 10.1371/journal.pone.0142887 (PMC4643966; doi:10.1371/journal.pone.0142887)

**S2 Fig. Enrichment profile of CACNA1A in human various cells/tissues**

**
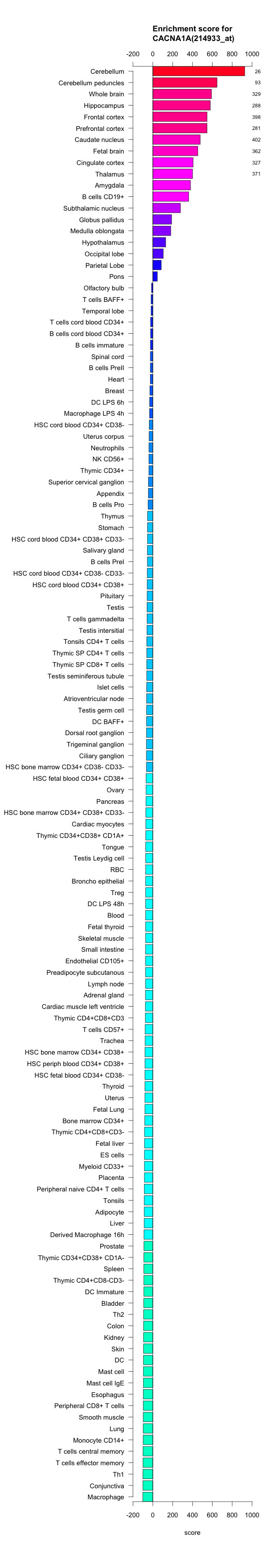
**

Supplement: S2 Fig — (DOC) [file pone.0142887.s002.doc]
