# Supplementary material for: Genetic Evidence for Possible Involvement of the Calcium Channel Gene CACNA1A in Autism Pathogenesis in Chinese Han Population
Source: PLoS One. 2015 Nov 13;10(11):e0142887. doi: 10.1371/journal.pone.0142887 (PMC4643966; doi:10.1371/journal.pone.0142887)
Supplement: S3 Fig — CBC, the cerebellar cortex; MD, mediodorsal nucleus of the thalamus; STR, striatum; AMY, amygdala; HIP, hippocampus; NCX, 11 areas of neocortex (DOC) [file pone.0142887.s003.doc]

**S3 Fig. Dynamic expression levels of CACNA1A in the human brain throughout life**


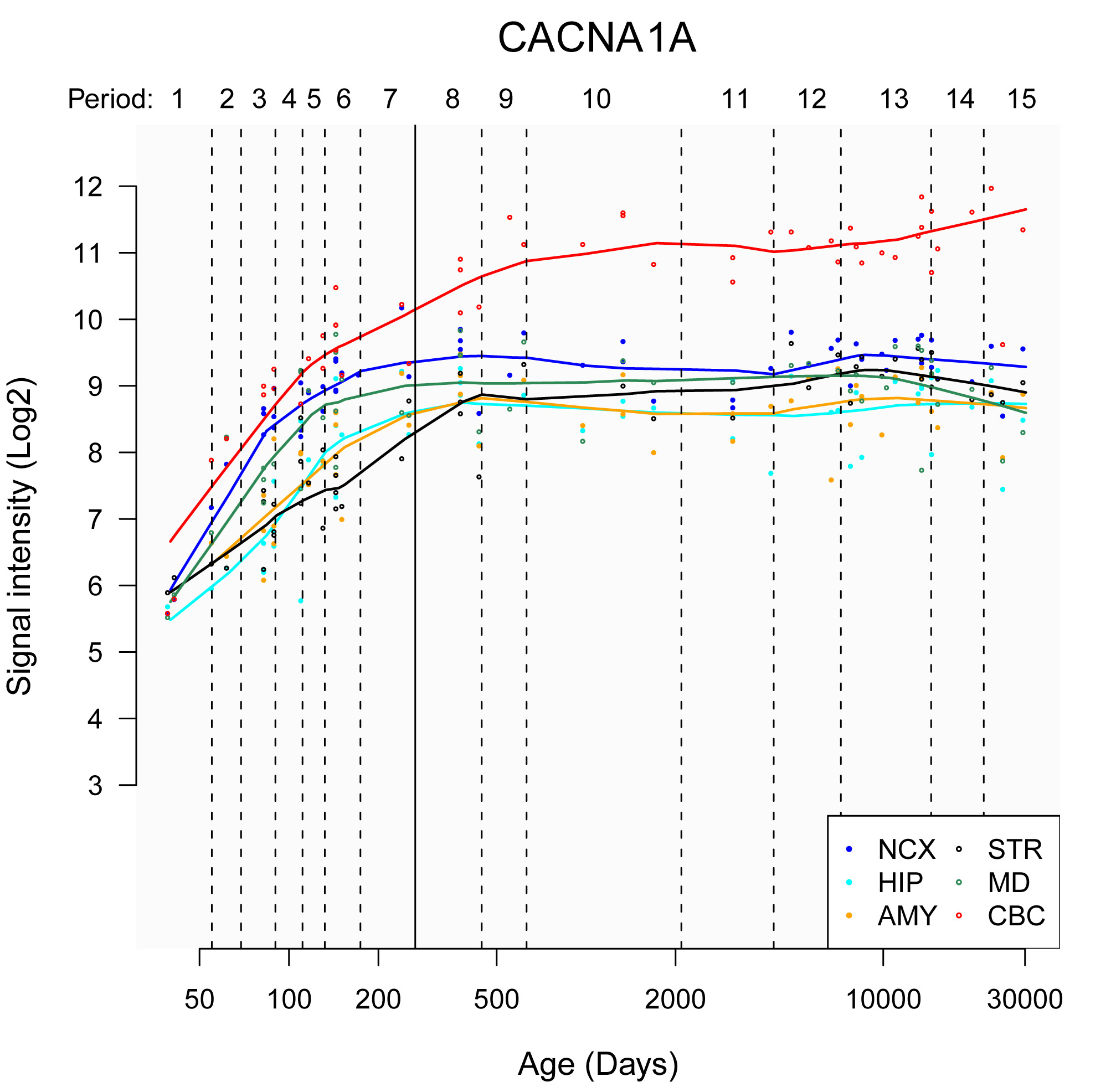


CBC, the cerebellar cortex; MD, mediodorsal nucleus of the thalamus; STR, striatum; AMY, amygdala; HIP, hippocampus; NCX, 11 areas of neocortex
