# Supplementary material for: Genetic Evidence for Possible Involvement of the Calcium Channel Gene CACNA1A in Autism Pathogenesis in Chinese Han Population
Source: PLoS One. 2015 Nov 13;10(11):e0142887. doi: 10.1371/journal.pone.0142887 (PMC4643966; doi:10.1371/journal.pone.0142887)
Supplement: S1 Table — a Hardy-Weinberg equilibrium p value for genotype distributions in children affected with autism; b Hardy-Weinberg equilibrium p value for genotype distributions in parents. (DOCX) [file pone.0142887.s004.docx]

**S1 Table.** **Information of the selected 12 SNPs in *CACNA1A* and genotype frequencies in 239 autism trios of Han Chinese descent**

| **Marker** | **Chr. position** | **Genotype frequencies in children** | | | ***p* _HWE_ ^a^** | **Genotype frequencies in parents** | | | ***p* _HWE_ ^b^** |
| --- | --- | --- | --- | --- | --- | --- | --- | --- | --- |
| rs7249246 | 13488269 | GG | GT | TT | 0.056 | GG | GT | TT | 0.103 |
|  |  | 47 | 134 | 58 |  | 122 | 221 | 135 |  |
| rs12609735 | 13477702 | CC | CT | TT | 0.921 | CC | CT | TT | 0.720 |
|  |  | 37 | 115 | 87 |  | 63 | 215 | 197 |  |
| rs10422148 | 13460497 | AA | AC | CC | 0.829 | AA | AC | CC | 0.076 |
|  |  | 76 | 119 | 44 |  | 166 | 215 | 97 |  |
| rs7252635 | 13442128 | CC | CT | TT | 0.283 | CC | CT | TT | 0.001 |
|  |  | 135 | 85 | 19 |  | 274 | 156 | 46 |  |
| rs10416717 | 13410714 | AA | AG | GG | 0.750 | AA | AG | GG | 0.720 |
|  |  | 59 | 117 | 63 |  | 117 | 235 | 126 |  |
| rs10425460 | 13330527 | AA | AC | CC | 0.538 | AA | AC | CC | 0.603 |
|  |  | 161 | 72 | 6 |  | 318 | 145 | 14 |  |
| rs1502017 | 13319510 | AA | AG | GG | 0.115 | AA | AG | GG | 0.133 |
|  |  | 15 | 108 | 115 |  | 29 | 202 | 245 |  |
| rs2419244 | 13311910 | AA | AG | GG | 0.062 | AA | AG | GG | 0.730 |
|  |  | 63 | 133 | 43 |  | 151 | 232 | 95 |  |
| rs8182538 | 13305356 | AA | AG | GG | 0.838 | AA | AG | GG | 0.454 |
|  |  | 62 | 121 | 56 |  | 109 | 247 | 122 |  |
| rs8104916 | 13287421 | CC | CT | TT | 0.103 | CC | CT | TT | 0.901 |
|  |  | 4 | 35 | 200 |  | 4 | 77 | 397 |  |
| rs11085838 | 13242908 | CC | CT | TT | 0.369 | CC | CT | TT | 0.097 |
|  |  | 31 | 119 | 89 |  | 81 | 210 | 187 |  |
| rs4926143 | 13214919 | CC | CT | TT | 0.295 | CC | CT | TT | 0.384 |
|  |  | 4 | 41 | 194 |  | 4 | 98 | 376 |  |

^a^ Hardy-Weinberg equilibrium *p* value for genotype distributions in children affected with autism; ^b^ Hardy-Weinberg equilibrium *p* value for genotype distributions in parents.
