# Supplementary material for: Genetic Evidence for Possible Involvement of the Calcium Channel Gene CACNA1A in Autism Pathogenesis in Chinese Han Population
Source: PLoS One. 2015 Nov 13;10(11):e0142887. doi: 10.1371/journal.pone.0142887 (PMC4643966; doi:10.1371/journal.pone.0142887)
Supplement: S2 Table — Afreq, allele frequency; Fam, number of informative families; S, test statistics for the observed number of transmitted alleles; E(S), expected value of S under the null hypothesis (i.e., no linkage and no association). (DOCX) [file pone.0142887.s005.docx]

**S2 Table. Results of association analyses between 12 SNPs in *CACNA1A* and autism in 239 trios by FBAT under an additive model**

| **Marker** | **Allele** | **Afreq** | **Fam** | **S** | **E (S)** | **Var (S)** | **Z** | ***p*** |
| --- | --- | --- | --- | --- | --- | --- | --- | --- |
| rs7249246 | G | 0.486 | 166 | 154.0 | 158.50 | 55.25 | -0.605 | 0.545 |
|  | T | 0.514 | 166 | 178.0 | 173.50 | 55.25 | 0.605 | 0.545 |
| **rs12609735** | C | 0.360 | 167 | 152.0 | 135.50 | 53.25 | 2.261 | **0.024** |
|  | T | 0.640 | 167 | 182.0 | 198.50 | 53.25 | -2.261 | **0.024** |
| rs10422148 | A | 0.570 | 168 | 181.0 | 182.50 | 53.75 | -0.205 | 0.838 |
|  | C | 0.430 | 168 | 155.0 | 153.50 | 53.75 | 0.205 | 0.838 |
| rs7252635 | C | 0.754 | 125 | 172.0 | 167.00 | 37.00 | 0.822 | 0.411 |
|  | T | 0.246 | 125 | 78.0 | 83.00 | 37.00 | -0.822 | 0.411 |
| rs10416717 | A | 0.489 | 174 | 170.0 | 168.00 | 58.50 | 0.261 | 0.794 |
|  | G | 0.511 | 174 | 178.0 | 180.00 | 58.50 | -0.261 | 0.794 |
| rs10425460 | A | 0.818 | 127 | 183.0 | 180.00 | 36.00 | 0.500 | 0.617 |
|  | C | 0.182 | 127 | 71.0 | 74.00 | 36.00 | -0.500 | 0.617 |
| rs1502017 | A | 0.276 | 159 | 119.0 | 113.00 | 50.50 | 0.844 | 0.398 |
|  | G | 0.724 | 159 | 199.0 | 205.00 | 50.50 | -0.844 | 0.398 |
| rs2419244 | A | 0.559 | 179 | 190.0 | 198.00 | 58.00 | -1.050 | 0.294 |
|  | G | 0.441 | 179 | 168.0 | 160.00 | 58.00 | 1.050 | 0.294 |
| rs8182538 | A | 0.484 | 186 | 196.0 | 182.50 | 61.75 | 1.718 | 0.086 |
|  | G | 0.516 | 186 | 176.0 | 189.50 | 61.75 | -1.718 | 0.086 |
| rs8104916 | C | 0.089 | 69 | 39.0 | 39.50 | 19.25 | -0.114 | 0.909 |
|  | T | 0.911 | 69 | 99.0 | 98.50 | 19.25 | 0.114 | 0.909 |
| rs11085838 | C | 0.390 | 169 | 140.0 | 146.50 | 52.25 | -0.899 | 0.369 |
|  | T | 0.610 | 169 | 198.0 | 191.50 | 52.25 | 0.899 | 0.369 |
| rs4926143 | C | 0.111 | 87 | 46.0 | 50.00 | 24.50 | -0.808 | 0.419 |
|  | T | 0.889 | 87 | 128.0 | 124.00 | 24.50 | 0.808 | 0.419 |

Afreq, allele frequency; Fam, number of informative families; S, test statistics for the observed number of transmitted alleles; E(S), expected value of S under the null hypothesis (i.e., no linkage and no association).
