# Supplementary material for: Genetic Evidence for Possible Involvement of the Calcium Channel Gene CACNA1A in Autism Pathogenesis in Chinese Han Population
Source: PLoS One. 2015 Nov 13;10(11):e0142887. doi: 10.1371/journal.pone.0142887 (PMC4643966; doi:10.1371/journal.pone.0142887)
Supplement: S3 Table — Afreq, allele frequency; Fam, number of informative families; S, test statistics for the observed number of transmitted alleles; E(S), expected value of S under the null hypothesis (i.e., no linkage and no association). (DOCX) [file pone.0142887.s006.docx]

**S3 Table. Results of association analyses between 12 SNPs in *CACNA1A* and autism in 239 trios by FBAT under a recessive model**

| **Marker** | **Allele** | **Afreq** | **Fam** | **S** | **E (S)** | **Var (S)** | **Z** | ***p*** |
| --- | --- | --- | --- | --- | --- | --- | --- | --- |
| **rs7249246** | G | 0.486 | 103 | 27.0 | 37.75 | 22.31 | -2.276 | **0.023** |
|  | T | 0.514 | 118 | 39.0 | 45.25 | 26.06 | -1.224 | 0.221 |
| **rs12609735** | C | 0.360 | 75 | 34.0 | 26.00 | 15.88 | 2.008 | **0.045** |
|  | T | 0.640 | 138 | 49.0 | 57.50 | 31.63 | -1.511 | 0.131 |
| rs10422148 | A | 0.570 | 122 | 48.0 | 49.25 | 27.56 | -0.238 | 0.812 |
|  | C | 0.430 | 93 | 35.0 | 34.75 | 20.31 | 0.055 | 0.956 |
| rs7252635 | C | 0.754 | 116 | 55.0 | 52.25 | 27.56 | 0.524 | 0.600 |
|  | T | 0.246 | 32 | 8.0 | 10.25 | 6.56 | -0.878 | 0.380 |
| rs10416717 | A | 0.489 | 111 | 45.0 | 40.50 | 24.00 | 0.919 | 0.358 |
|  | G | 0.511 | 123 | 49.0 | 46.50 | 27.00 | 0.481 | 0.630 |
| rs10425460 | A | 0.818 | 125 | 62.0 | 58.25 | 30.19 | 0.683 | 0.495 |
|  | C | 0.182 | 19 | 6.0 | 5.25 | 3.69 | 0.391 | 0.696 |
| rs1502017 | A | 0.276 | 55 | 14.0 | 16.75 | 11.06 | -0.827 | 0.408 |
|  | G | 0.724 | 147 | 54.0 | 62.75 | 34.06 | -1.499 | 0.134 |
| **rs2419244** | A | 0.559 | 135 | 43.0 | 54.25 | 30.44 | -2.039 | **0.041** |
|  | G | 0.441 | 97 | 32.0 | 35.25 | 20.94 | -0.710 | 0.478 |
| rs8182538 | A | 0.484 | 120 | 52.0 | 44.75 | 26.19 | 1.417 | 0.157 |
|  | G | 0.516 | 127 | 42.0 | 48.25 | 27.94 | -1.182 | 0.237 |
| rs8104916 | C | 0.089 | 9 | - | - | - | - | - |
|  | T | 0.911 | 68 | 34.0 | 32.00 | 16.50 | 0.492 | 0.622 |
| rs11085838 | C | 0.390 | 82 | 28.0 | 31.00 | 18.00 | -0.707 | 0.480 |
|  | T | 0.610 | 127 | 57.0 | 53.50 | 29.25 | 0.647 | 0.518 |
| rs4926143 | C | 0.111 | 12 | 4.00 | 3.25 | 2.31 | 0.439 | 0.622 |
|  | T | 0.889 | 86 | 45.00 | 40.25 | 20.81 | 1.041 | 0.298 |

Afreq, allele frequency; Fam, number of informative families; S, test statistics for the observed number of transmitted alleles; E(S), expected value of S under the null hypothesis (i.e., no linkage and no association).
