# Supplementary material for: Genetic Evidence for Possible Involvement of the Calcium Channel Gene CACNA1A in Autism Pathogenesis in Chinese Han Population
Source: PLoS One. 2015 Nov 13;10(11):e0142887. doi: 10.1371/journal.pone.0142887 (PMC4643966; doi:10.1371/journal.pone.0142887)
Supplement: S4 Table — Afreq, allele frequency; Fam, number of informative families; S, test statistics for the observed number of transmitted alleles; E(S), expected value of S under the null hypothesis (i.e., no linkage and no association). (DOCX) [file pone.0142887.s007.docx]

**S4 Table. Results of association analyses between 12 SNPs in *CACNA1A* and autism in 239 trios by FBAT under a dominant model**

| **Marker** | **Allele** | **Afreq** | **Fam** | **S** | **E (S)** | **Var (S)** | **Z** | ***p*** |
| --- | --- | --- | --- | --- | --- | --- | --- | --- |
| **rs7249246** | G | 0.486 | 118 | 79.0 | 72.75 | 26.06 | 1.224 | 0.221 |
|  | T | 0.514 | 103 | 76.0 | 65.25 | 22.31 | 2.276 | **0.023** |
| **rs12609735** | C | 0.360 | 138 | 89.0 | 80.50 | 31.63 | 1.511 | 0.131 |
|  | T | 0.640 | 75 | 41.0 | 49.00 | 15.88 | -2.008 | **0.045** |
| rs10422148 | A | 0.570 | 93 | 58.0 | 58.25 | 20.31 | -0.055 | 0.956 |
|  | C | 0.430 | 122 | 74.0 | 72.75 | 27.56 | 0.238 | 0.812 |
| rs7252635 | C | 0.754 | 32 | 24.0 | 21.75 | 6.56 | 0.878 | 0.380 |
|  | T | 0.246 | 116 | 61.0 | 63.75 | 27.56 | -0.524 | 0.600 |
| rs10416717 | A | 0.489 | 123 | 74.0 | 76.50 | 27.00 | -0.481 | 0.630 |
|  | G | 0.511 | 111 | 66.0 | 70.50 | 24.00 | -0.919 | 0.358 |
| rs10425460 | A | 0.818 | 19 | 13.0 | 13.75 | 3.69 | -0.391 | 0.696 |
|  | C | 0.182 | 125 | 63.0 | 66.75 | 30.19 | -0.683 | 0.495 |
| rs1502017 | A | 0.276 | 147 | 93.0 | 84.25 | 34.06 | 1.499 | 0.134 |
|  | G | 0.724 | 55 | 41.0 | 38.25 | 11.06 | 0.827 | 0.408 |
| **rs2419244** | A | 0.559 | 97 | 65.0 | 61.75 | 20.94 | 0.710 | 0.478 |
|  | G | 0.441 | 135 | 92.0 | 80.75 | 30.44 | 2.039 | **0.041** |
| rs8182538 | A | 0.484 | 127 | 85.0 | 78.75 | 27.94 | 1.182 | 0.237 |
|  | G | 0.516 | 120 | 68.0 | 75.25 | 26.19 | -1.417 | 0.157 |
| rs8104916 | C | 0.089 | 68 | 34.0 | 36.00 | 16.50 | -0.492 | 0.622 |
|  | T | 0.911 | 9 | - | - | - | - | - |
| rs11085838 | C | 0.390 | 127 | 70.0 | 73.50 | 29.25 | -0.647 | 0.518 |
|  | T | 0.610 | 82 | 54.0 | 51.00 | 18.00 | 0.707 | 0.480 |
| rs4926143 | C | 0.111 | 86 | 41.0 | 45.75 | 20.81 | -1.041 | 0.298 |
|  | T | 0.889 | 12 | 8.0 | 8.75 | 2.31 | -0.439 | 0.622 |

Afreq, allele frequency; Fam, number of informative families; S, test statistics for the observed number of transmitted alleles; E(S), expected value of S under the null hypothesis (i.e., no linkage and no association).
