# Supplementary material for: Genetic Evidence for Possible Involvement of the Calcium Channel Gene CACNA1A in Autism Pathogenesis in Chinese Han Population
Source: PLoS One. 2015 Nov 13;10(11):e0142887. doi: 10.1371/journal.pone.0142887 (PMC4643966; doi:10.1371/journal.pone.0142887)
Supplement: S5 Table — a Hardy-Weinberg equilibrium p value for genotype distributions in children affected with autism; b Hardy-Weinberg equilibrium p value for genotype distributions in parents. (DOCX) [file pone.0142887.s008.docx]

**S5 Table.** **Information of 3 SNPs in *CACNA1A* and genotype frequencies in 553 autism trios of Han Chinese descent**

| **Marker** | **Chr. position** | **Genotype frequencies in children** | | | ***p* _HWE_ ^a^** | **Genotype frequencies in parents** | | | ***p* _HWE_ ^b^** |
| --- | --- | --- | --- | --- | --- | --- | --- | --- | --- |
| rs7249246 | 13488269 | GG | GT | TT | 0.031 | GG | GT | TT | 0.628 |
|  |  | 116 | 297 | 131 |  | 278 | 536 | 274 |  |
| rs12609735 | 13477702 | CC | CT | TT | 0.407 | CC | CT | TT | 0.656 |
|  |  | 67 | 260 | 216 |  | 132 | 483 | 469 |  |
| rs2419244 | 13311910 | AA | AG | GG | 0.452 | AA | AG | GG | 0.957 |
|  |  | 160 | 278 | 106 |  | 343 | 535 | 210 |  |

^a^ Hardy-Weinberg equilibrium *p* value for genotype distributions in children affected with autism;

^b^ Hardy-Weinberg equilibrium *p* value for genotype distributions in parents.
